# Whole-Genome Resequencing Reveals Deep Genomic Differentiation and Highly Differentiated Segments Between a Composite Domestic Cattle Population and Yak from the Ili River Valley and Other Xinjiang Regions

**DOI:** 10.3390/ani16111746

**Published:** 2026-06-05

**Authors:** Guzalnur Amat, Bo Hu, Yong Tuo, Adiljan Kader, Ablat Sulayman, Zhenghong Zhan, Jianping Zhu, Zhijun Zhang, Bayin Bate, Ziyi Ren, Amat Mamat, Akida Tursun, Tongjun Guo

**Affiliations:** 1 Xinjiang Key Laboratory of Feed Biotechnology, Urumqi 830011, China; mamatali6289@126.com (G.A.); t777yvx@126.com (Y.T.); 19990316678@163.com (Z.Z.); 13579224807@163.com (Z.Z.); 13899868383@163.com (B.B.); renziyi0505@163.com (Z.R.); 15022981775@163.com (A.T.); 2 Institute of Feed Research, Xinjiang Academy of Animal Science, Urumqi 830011, China; 3 Institute of Animal Husbandry, Xinjiang Academy of Animal Science, Urumqi 830011, China; 15352627527@163.com (B.H.); iblat2009@sina.cn (A.S.); xmaind@126.com (A.M.); 4 College of Life Science, Xinjiang Agricultural University, Urumqi 830052, China; adilbj@126.com; 5 Tacheng Prefecture Animal Husbandry Science and Technology Research and Extension Center, Tacheng 834700, China; 18095958045@163.com

**Keywords:** domestic cattle, yak, Xinjiang, population genomics, weighted F_ST, highly differentiated segments, Tajima’s D, linkage disequilibrium, non-tree-like covariance, PSMC

## Abstract

The Ili River Valley and adjacent Xinjiang regions contain introduced cattle, local cattle, and yak and therefore provide a useful regional system for examining cattle–yak genomic differentiation. Using whole-genome resequencing data from 79 individuals, we analyzed Angus (ANG), Simmental (SIM), Holstein (HOL), Xinjiang Brown Cattle (XH), Kazakh Cattle (KAZ), Altay White-headed Cattle (AWH), and yak (WY). The six domestic cattle groups were merged into a composite domestic cattle group (PTN, *n* = 69) and compared with WY (*n* = 10). Sequencing generated 2996.28 Gb of raw data and 2939.56 Gb of clean data. Alignment to the Bos taurus ARS-UCD1.2 reference genome yielded mapping rates of 96.71–99.78% and depths of 5.98×–17.26×. Genome-wide PTN-WY comparisons showed extremely high differentiation: the median weighted F_ST was 0.846 and the 95th percentile was 0.943. The joint F_ST–π scan identified 832 candidate highly differentiated windows and 533 unique ENSBTAG gene IDs, whereas the low-differentiation set contained only five windows and three genes. The longest contiguous highly differentiated segments were located on chromosomes 26, 29, 8, 21, and 7. WY had the highest median Tajima’s D (1.173) and the slowest LD decay, while KAZ had the lowest median Tajima’s D (0.345) and the fastest LD decay. Treemix supported non-tree-like covariance components, and PSMC indicated broadly similar deep-time demographic profiles across individuals. Overall, the dominant genomic signal between PTN and WY is deep phylogenetic divergence, with locally enhanced highly differentiated segments superimposed on this background. These segments were enriched for functions and pathways related to reproductive behavior, neuroendocrine regulation, circadian rhythm, and membrane transport, but they are not interpreted here as recent within-species selective sweeps. The results provide a cautious regional framework for conservation and breeding of bovine genetic resources in Xinjiang.

## Simple Summary

The Ili River Valley and adjacent Xinjiang regions contain introduced cattle breeds, local cattle breeds, and yak. Using whole-genome resequencing of 79 animals, we compared a composite domestic cattle group (PTN) with yak (WY) under a unified cattle-reference coordinate system. The dominant signal was deep cattle–yak genomic divergence, with multiple locally enhanced highly differentiated segments superimposed on this background. These segments were enriched for interconnected functions related to reproductive behavior, neuroendocrine regulation, circadian rhythm, and membrane transport. Yak also showed distinct Tajima’s D, LD, and broad PSMC patterns. This study provides a regional genomic framework for interpreting cattle–yak differentiation and for conserving bovine genetic resources in Xinjiang.

## 1. Introduction

The evolution of modern domestic cattle cannot be attributed to a single domestication event. Mitochondrial and whole-genome studies indicate that common cattle and zebu cattle correspond to at least two major domestication processes, followed by continuous migration, hybridization, local adaptation, and breed formation [1,2,3,4,5,6,7,8]. In East Asia, this process is even more complex, as local cattle populations have been influenced not only by ancient domestication lineages but also by historical events such as the introduction of foreign breeds, local selective breeding, and genetic admixture with closely related bovine taxa [3,7,8,9,10,11]. Consequently, signals of high genetic differentiation within populations are often not the product of a single mechanism, but rather the result of the combined effects of phylogenetic divergence, genetic drift, local selection, and historical admixture.

Yaks occupy a unique position within the genus *Bos*. Previous studies have shown that yaks and domestic cattle exhibit deep phylogenetic divergence, with their genomes retaining a large number of specific signals associated with high-altitude, hypoxic environments, and energy metabolism [9,12,13,14]. On the other hand, yak and domestic cattle are not completely isolated; multiple studies have detected limited but identifiable historical gene flow [9,10,13]. Therefore, the genomic pattern of “coexisting extensive differentiation and local admixture” observed in regions where yak and domestic cattle coexist has clear biological plausibility.

The Ili River Valley and adjacent Xinjiang regions, where commercially introduced cattle, local cattle, and yak coexist within a broader regional production system, provide an informative setting for studying bovine differentiation and historical processes. In this study, using a unified reference-genome coordinate system, we conducted F_ST, π, Tajima’s D, LD decay, Treemix, PSMC, and GO/KEGG enrichment analyses on 79 individuals to address three questions: whether the differences between the composite domestic cattle population and yak are genome-wide or concentrated in locally enhanced highly differentiated segments; whether these segments form interpretable functional networks; and to what extent the observed differentiation pattern is associated with broad population history and non-tree-like covariance. Breed-level population structure in the same regional system has been analyzed separately in our recent seven-population study [15], whereas the present manuscript is intentionally centered on the PTN-WY comparison.

## 2. Materials and Methods

### 2.1. Study Populations and Sequencing Data

A total of 79 individuals were sampled from seven bovine groups: Angus (ANG, *n* = 8), Simmental (SIM, *n* = 7), Holstein (HOL, *n* = 8), Xinjiang Brown Cattle (XH, *n* = 12), Kazakh Cattle (KAZ, *n* = 19), Altay White-headed Cattle (AWH, *n* = 15), and yak (WY, *n* = 10). ANG, SIM, HOL, XH, KAZ, and AWH were collected from herds maintained in the Ili River Valley of Xinjiang, whereas WY was sampled from Kizilsu Prefecture in Xinjiang, consistent with the regional sampling framework reported in our previous seven-population study [15]. ANG, SIM, and HOL therefore represent introduced commercial breeds maintained under local production conditions in the Ili region rather than reference populations from Europe or North America. Because detailed herd-by-herd introduction histories were not uniformly available for all farms, we did not convert residence history into exact years or generations; this limitation is acknowledged explicitly in the revised Discussion. To highlight the overall cattle–yak contrast, the six domestic cattle groups were merged into a composite domestic cattle group (PTN, *n* = 69), while WY was retained separately. Subsequent analyses therefore describe the dominant PTN-WY differentiation pattern rather than breed-specific signals from any single domestic breed. Consequently, PTN is an intentionally heterogeneous composite background; therefore, the within-group nucleotide diversity (π_PTN) is expected to be inflated, and the PTN-WY F_ST distribution may be broadened by intra-PTN structure. The candidate highly differentiated segments identified in this study are thus interpreted as composite domestic-cattle-versus-yak contrast regions rather than as breed-specific signals.

Sequencing yielded a total of 2996.28 Gb of raw data, which was filtered to retain 2939.56 Gb of clean data. Per-sample raw data ranged from 20.15 to 58.13 Gb, and clean data ranged from 20.14 to 57.36 Gb; the effective coverage ranged from 87.01% to 99.94%, Q20 ranged from 95.14% to 99.32%, Q30 from 88.93% to 97.09%, and GC content from 43.11% to 49.40%.

### 2.2. Reference Genome, Read Processing, Alignment, and Variant Detection

In this study, all clean reads from all samples were aligned to the Bos taurus reference genome ARS-UCD1.2 (Ensembl release 109) [16]. This reference genome contains 2211 sequences with a total length of 2,715,853,792 bp, a GC content of 41.93%, a gap rate of 0.00%, and N50 and N90 values of 103,308,737 bp and 51,098,607 bp, respectively.

Raw sequencing data were quality controlled using fastp v0.20.0 with the key parameters -g -q 5 -u 50 -n 15 -l 150. To ensure that subsequent comparisons were performed in a unified coordinate system, the filtered clean reads were aligned to the reference genome using BWA-MEM v0.7.17-r1188 (mem -t 4 -k 32 -M) [17]. Duplicate reads were removed using SAMtools [18]. Variants were then called with bcftools v1.16 using the verified archived parameters mpileup -q 1 -C 50 -a DP, SP, AD -m 2 -F 0.002 [19]. During SNP detection and initial screening, loci supported by at least four reads and with mapping quality (MQ) ≥ 20 were retained. Variant functional annotation was performed using ANNOVAR (version 14 December 2015) [20]. Because the retained archived workflow does not support additional verified claims about global MAF filters, missing-rate filters, biallelic-only pruning, or low-complexity masking across every downstream module, we report only those filtering parameters that could be directly confirmed from the preserved workflow records.

F_ST, π, and Tajima’s D were calculated using VCFtools v0.1.16 [21]; LD decay was calculated using PopLDdecay v3.40 with the key parameter -OutStat [22]; Treemix and PSMC were run using v1.12 and v0.6.4-r49, respectively [23,24]. The alignment rates for the 79 samples ranged from 96.71% to 99.78%, with an average sequencing depth of 5.98×–17.26×. Coverage ≥ 1× ranged from 82.96% to 98.68%, and coverage ≥ 4× ranged from 64.10% to 97.91%, indicating that the data were adequate for downstream window-based population genomic analysis. Because all samples, including yak, were mapped to the Bos taurus reference genome, cross-species comparisons may be influenced by reference bias through differential mappability, callable sites, local mismatch rates, and potentially downward-biased yak diversity estimates [25]. These effects are considered explicitly in the interpretation of π, F_ST, and LD-related results. To improve revision-stage reproducibility, the recoverable confirmed parameters and preserved archived outputs for each analytical module are consolidated in Supplementary Table S1. By contrast, cluster-level duplicate rates, Ti/Tv values, and some pre- and post-filter SNP-count logs were not recoverable from the surviving archive and are therefore not reconstructed here as inferred values.

### 2.3. F_ST, π, and Candidate Window Recognition

**Figure 1:**
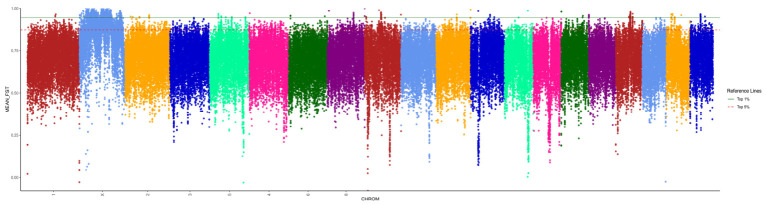
Distribution of the genome-wide MEAN_FST between PTN and WY. The sliding-window MEAN_FST was calculated using a 40 kb window and a 20 kb step size; the dashed lines indicate empirical quantile thresholds. This figure illustrates the genome-wide distribution of divergence between PTN and WY; candidate windows are still identified based on the joint extremes of the WEIGHTED_FST and π ratios.

**Figure 2:**
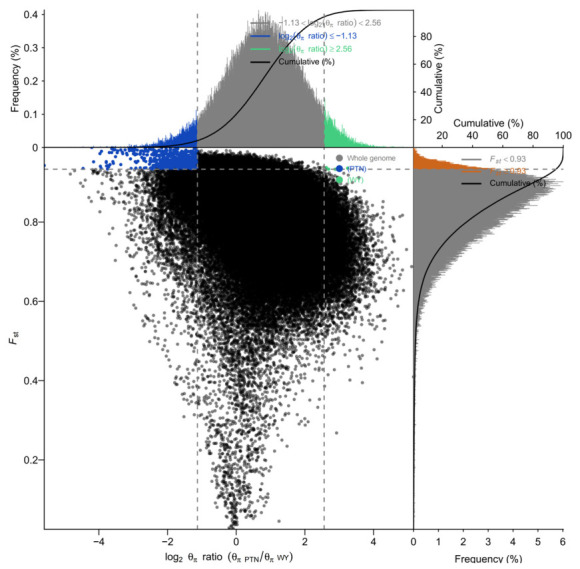
Joint WEIGHTED_F_ST-log2(π_PTN/π_WY) screening plot for PTN and WY. Candidate highly differentiated windows were defined as windows simultaneously located in the high-F_ST tail (WEIGHTED_F_ST ≥ 0.93) and an extreme π-ratio tail (log2(π_PTN/π_WY) ≤ −1.13 or ≥2.58). The dashed lines indicate the empirical thresholds for WEIGHTED_F_ST (0.93) and log2(π_PTN/π_WY) (−1.13 and 2.58).

Sliding-window analyses of F_ST and π were performed using 40 kb windows and 20 kb steps. This window-step combination was chosen as a compromise between chromosome-scale smoothing and retention of local heterogeneity in a dataset with moderate sequencing depth and genome-wide SNP density. Figure 1 visualizes the genome-wide landscape using MEAN_FST, whereas candidate-window ranking and the headline numerical thresholds in the Abstract and Results are based on WEIGHTED_FST because the weighted estimator is less sensitive to uneven SNP counts among windows. The joint F_ST–π scan was conducted using WEIGHTED_FST together with the empirical distribution of log2(π_PTN/π_WY). In the archived screening plot (Figure 2), the extreme π-ratio tails were defined as log2(π_PTN/π_WY) ≤ −1.13 or ≥2.58, and the high-F_ST tail was defined as WEIGHTED_FST ≥ 0.93. Windows simultaneously located in the high-F_ST tail and an extreme π-ratio tail were treated as candidate highly differentiated windows. Windows at the opposite joint extreme were retained as the bottom candidate set. These categories are used here to describe locally highly differentiated versus relatively conserved segments rather than cattle-specific or yak-specific recent selection signatures.

### 2.4. Tajima’s D

Tajima’s D is calculated separately for each population using a 40 kb window to describe the deviation of the allele frequency profile within a population from the neutral model [26]. In this study, it is used as a descriptive indicator of differences in frequency profiles and population history, rather than treating a single extreme value of Tajima’s D as direct evidence of selection.

### 2.5. LD Decay

Linkage disequilibrium decay was calculated using PopLDdecay v3.40, with the key parameter set to -OutStat [22]. The decay process of haplotype blocks was described using the average r^2^ across different distance intervals. LD decay is primarily used to help interpret differences in effective population size and linkage structure.

### 2.6. Treemix Non-Phylogenetic Analysis

Treemix v1.12 constructs maximum-likelihood trees based on genome-wide allele-frequency data and improves covariance fitting by incorporating migration edges [23]. The analysis used 19,281,978 SNPs, a block size of 1000, and models with m = 1, 2, and 3 migration edges. No explicit outgroup/root was set, and no LD pruning was applied before the archived Treemix run. Given these settings and the presence of linkage disequilibrium across the genome, the Treemix output is interpreted conservatively as evidence that covariance among the seven groups is not fully captured by a single bifurcating tree. We do not use it to make quantitative claims about the direction or magnitude of gene flow.

### 2.7. PSMC

PSMC v0.6.4-r49 infers historical effective population size from a single diploid genome [24]. Separate models were built for 79 individuals using the archived time-scale settings g = 5 and μ = 0.1 × 10^−8^, consistent with the original PSMC plot output retained for this project. Runtime parameters were -N30 -t15 -r5 -p 4 + 25*2 + 4 + 6. When constructing the PSMC input file, the samtools mpileup stage used -q 1 and -C 50; the vcfutils.pl vcf2fq stage used -d 5 -D 200 and -Q 20; and fq2psmcfa used -q10. No bootstrapping was performed. Because PSMC is sensitive to coverage variation and population structure and has limited power for very recent demographic events, the curves are used here only to compare broad macro-demographic trends rather than fine recent fluctuations or subtle between-population differences.

### 2.8. Functional Enrichment Analysis

GO and KEGG enrichment analyses for genes corresponding to the candidate highly differentiated and low-differentiated windows were performed using clusterProfiler in the R environment (version 4.6.0) [27]; information on the GO and KEGG databases was referenced from [28,29,30], respectively. Enrichment tests were based on the hypergeometric distribution, with Benjamini–Hochberg correction applied for multiple comparisons [31]. Enrichment was considered significant if the corrected *p*-value was <0.05. The background gene set was defined as all genes included in the sliding-window analysis that had valid annotations. The main text presents only representative significant entries; no manual removal of original significant results was performed.

## 3. Results

### 3.1. Sequencing Quality and Alignment Statistics Support Subsequent Whole-Genome Analysis

The sequencing data quality for the 79 individuals was generally stable. The total volume of raw data was 2996.28 Gb, and the total volume of clean data was 2939.56 Gb (Table 1). The average effective coverage rate for the samples was 98.07%, with average Q20 and Q30 values of 98.77% and 95.63%, respectively. The GC content was concentrated between 43.11% and 49.40%, with no abnormal outliers observed (Table 2).

Alignment results indicate a high degree of consistency between the samples and the reference genome. The average alignment rate for all samples was 99.48%, with an average sequencing depth of 11.69×. The average coverage at ≥1× and ≥4× was 97.97% and 92.05%, respectively. These metrics indicate that the data from the current 79 individuals is sufficient to support subsequent sliding-window statistics and population relationship analysis.

### 3.2. PTN and WY Exhibit Extremely High Genome-Wide Divergence, with the Primary Signal Reflecting Differences at the Phylogenetic Scale

The F_ST sliding-window analysis of PTN and WY yielded a total of 132,938 windows (Table 3). The first quartile of MEAN_FST was 0.617864, the median was 0.705037, the third quartile was 0.781816, the 95th percentile was 0.871117, and the 99th percentile was 0.945063. This distribution indicates that the background divergence in the PTN–WY comparison is already very high; therefore, the high-tail windows primarily reflect segments of high divergence across lineages. It should be noted that the F_ST distribution statistics reported in this section are used to describe the genome-wide differentiation pattern, while candidate window identification is still based on the joint extremes of the WEIGHTED_FST and π ratios.

The genomic distribution of F_ST exhibits a pattern of “widespread high differentiation + localized clustering enhancement.” The set of candidate highly divergent windows is most concentrated on chromosome X (97 windows), followed by chromosome 8 (54), chromosome 7 (51), chromosome 26 (44), chromosome 1 (43), and chromosome 2 (42). These results indicate that the divergence between domestic cattle and yak is genome-wide, but certain chromosomes carry more concentrated and continuous signals of divergence.

### 3.3. The Set of Candidate Highly Differentiated Windows Forms Multiple Contiguous Highly Differentiated Segments

The set of candidate highly differentiated windows contains 832 windows, of which 505 are annotated with genes, corresponding to a total of 533 unique ENSBTAG gene IDs. The weighted F_ST values in this set range from 0.9333 to 0.9881, with a mean of 0.9527. The low-differentiation candidate window set contains only five windows and three genes (Table 4). Since the tails on both sides are not strictly symmetrical in statistical terms, this numerical disparity is not directly used to infer the strength of selection on either side.

After merging based on window contiguity, the longest contiguous highly divergent segments are located on: chromosome 26 at 0.02–0.52 Mb (24 windows), chromosome 29 at 0.04–0.50 Mb (21 windows), chromosome 8 at 79.06–79.36 Mb (14 windows), chromosome 21 at 31.40–31.68 Mb (12 windows), and chromosome 7 at 49.98–50.18 Mb (9 windows). These segments reflect sustained local high differentiation rather than isolated anomalies.

### 3.4. Genomic Heterogeneity of π Landscapes

The π sliding-window analysis yielded a total of 132,332 windows (Table 5). The median π value across the entire genome was 0.00038477, with a mean of 0.00050616, a 95th percentile of 0.00132583, and a maximum of 0.0111174. The vast majority of windows exhibited low-to-moderate diversity levels, with only a small number maintaining higher π values.

**Figure 3:**
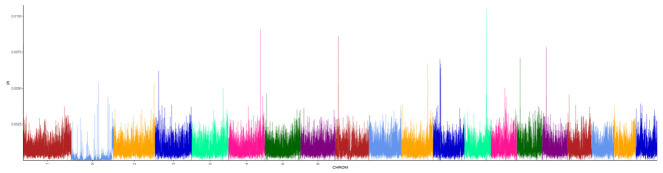
Genome-wide distribution of π in the PTN-WY comparison. Each distinct color represents a different chromosome, ordered sequentially from chromosome 1 to chromosome 29 and chromosome X.

The genomic distribution of π reveals that the troughs in diversity do not fully coincide with the peaks in F_ST. This indicates that the high divergence in PTN–WY is not entirely driven by a decline in local diversity. An explanation more consistent with the data is that deep phylogenetic divergence forms the primary background, upon which changes in local segment diversity and enhanced divergence are superimposed (Figure 3).

### 3.5. Tajima’s D Indicates Systematic Differences in the Frequency Spectra Between Yak and Domestic Cattle

The median values of Tajima’s D for each population were as follows: WY 1.173, HOL 0.695, ANG 0.606, AWH 0.443, SIM 0.437, XH 0.437, and KAZ 0.345. Among these, the first quartile for WY was 0.373, higher than that of all domestic cattle populations; the first quartile for KAZ was −0.429, with the most pronounced negative tail.

**Figure 4:**
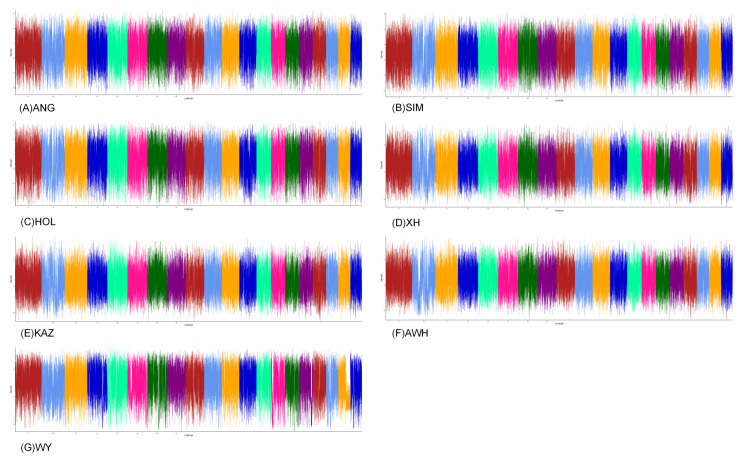
Genome-wide distribution of Tajima’s D across seven populations. (**A**) ANG; (**B**) SIM; (**C**) HOL; (**D**) XH; (**E**) KAZ; (**F**) AWH; (**G**) WY. The alternating colors represent distinct chromosomes aligned sequentially along the bovine genome coordinate system..

The proportions of negative-value windows in each population were as follows: WY 17.2%, HOL 27.0%, ANG 28.2%, SIM 34.0%, AWH 35.1%, XH 35.7%, and KAZ 38.4%. Conversely, the proportion of windows with D > 2 reached 20.3% in WY, significantly higher than in the other cattle populations (Table 6). These results indicate that the allele frequency spectrum of yak is generally biased toward medium- and high-frequency variants, whereas cattle populations retain more low-frequency variants. This pattern is consistent with the long-term population history and degree of genetic structuring among different populations (Figure 4).

### 3.6. LD Decay Patterns Distinguish Yak from Domestic Cattle and Local Cattle from Commercial Cattle

LD decay analysis revealed that WY consistently maintained the highest r^2^ across the entire 0–300 kb range and exhibited the slowest decay. KAZ had the lowest r^2^ and the fastest decay; AWH and XH were on the lower LD side; HOL and SIM maintained relatively higher r^2^ at medium to long distances, while ANG occupied an intermediate position.

**Figure 5:**
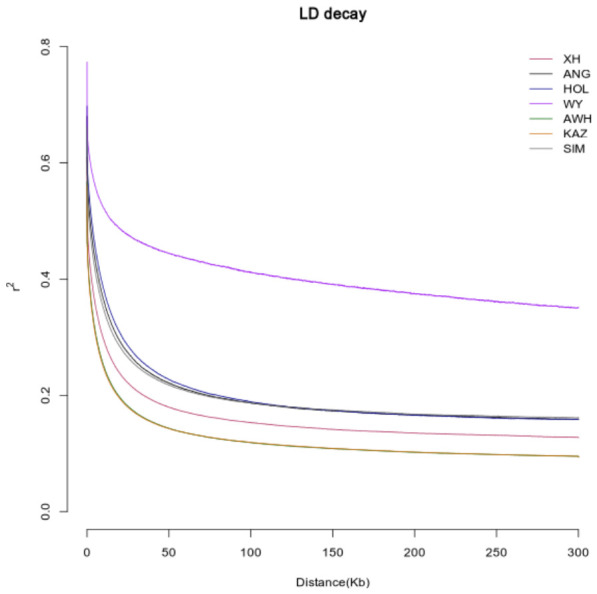
LD decay curves for the seven groups. The horizontal axis indicates physical distance, and the vertical axis indicates mean r^2^. WY retained the highest r^2^ across 0–300 kb, whereas KAZ showed the fastest decay.

This ranking aligns with the direction of population differences in Tajima’s D. The high LD in WY corresponds to its more positive distribution of Tajima’s D, indicating a smaller effective population size over the long term and longer haplotype blocks; KAZ, in contrast, exhibited faster haplotype fragmentation and a higher proportion of low-frequency variants (Figure 5).

### 3.7. Treemix Indicates That a Single-Branching Tree Is Insufficient to Explain Population Relationships

Treemix showed the main divergence pattern between yak and domestic cattle under migration edge models with m = 1, 2, and 3; however, the residual heatmap indicated that a strictly branching tree could not fully fit the covariance structure among the seven populations. The residual scale was ±24.1 SE when m = 1; it contracted to ±15.7 SE and ±15.8 SE when m = 2 and m = 3, respectively (Table 7). This result indicates that model fit improved significantly when non-tree-like components were allowed.

**Figure 6:**
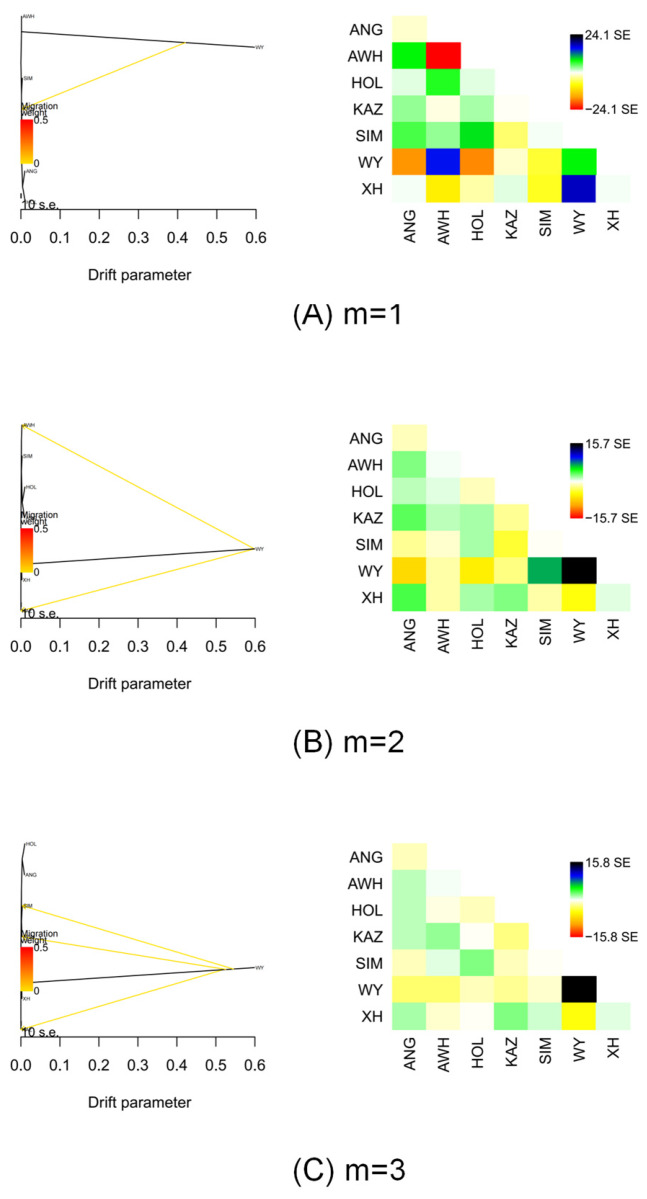
Treemix clustering diagram and residual heatmap. (**A**) m = 1; (**B**) m = 2; (**C**) m = 3.The colored arrows (yellow/orange) represent inferred migration edges, indicating pathways of historical genetic admixture or gene flow among the bovine groups. The horizontal scale bar beneath the tree represents the drift parameter, and the colored vertical scale bar indicates the migration weight.

Under the current settings, the robust conclusion is that covariance among the seven groups is not fully captured by a single bifurcating tree; no directional gene-flow claim is made here (Figure 6).

### 3.8. Functional Enrichment of the Candidate Highly Differentiated Window Set Is Concentrated in Reproductive Behavior, Neuroendocrine, and Membrane Transport Networks

The GO enrichment results for genes corresponding to the candidate highly differentiated window set comprise a total of 1644 terms, of which 155 are statistically significant after correction, including 106 biological processes, 37 molecular functions, and 12 cellular components. The most significant terms are concentrated in processes related to reproductive behavior, including multicellular organismal reproductive behavior, post-mating behavior, regulation of female receptivity, female mating behavior, mating behavior, mating, and reproductive behavior, all with adjusted *p*-values in the 10^−6^ range. At the molecular function level, drug binding, protein kinase binding, and kinase binding also appear among the significant terms.

**Figure 7:**
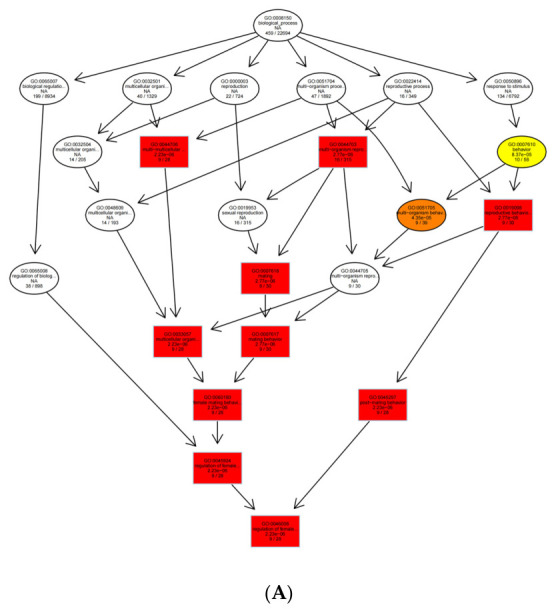

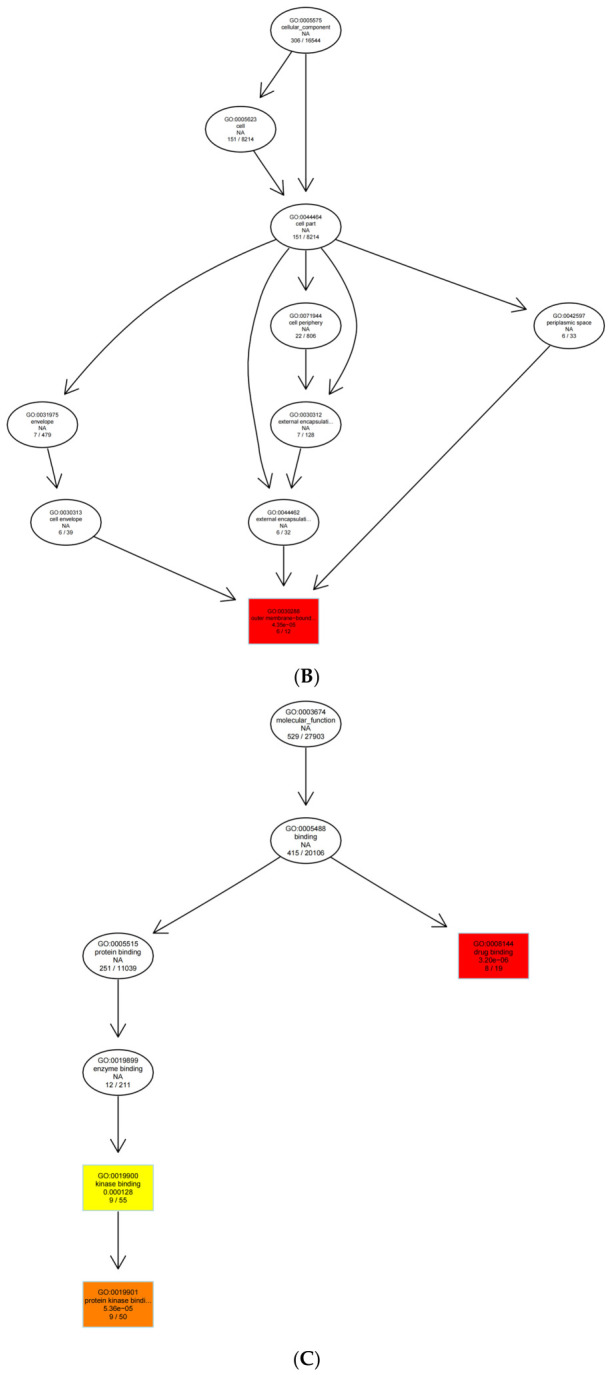

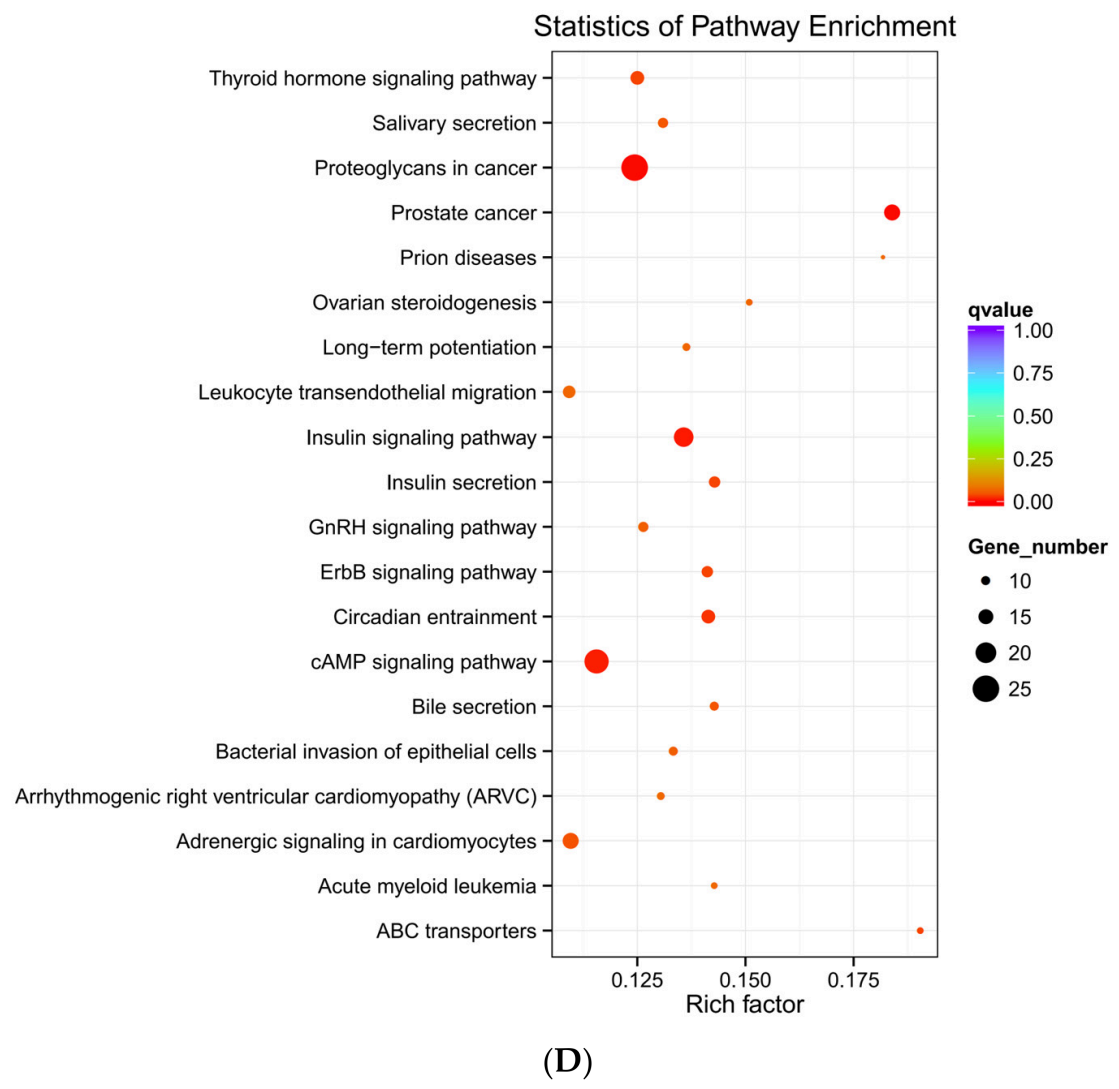
(**A**) DAG of the candidate highly differentiated window gene set for GO biological processes; (**B**) DAG of the candidate highly differentiated window gene set “GO cellular component.” (**C**) GO molecular function DAG for the set of candidate highly differentiated window genes; (**D**) KEGG enrichment scatter plot for the set of candidate highly differentiated window genes.(**A**–**C**), rectangular and elliptical nodes represent specific GO terms, where the color shading reflects the level of statistical significance: red indicates the most highly significant enrichment, followed by orange and yellow for intermediate significance, while white nodes represent non-significant or background terms required to preserve the hierarchical tree topology. In the scatter plot (**D**), the dot size corresponds to the gene number and the color scale represents the q-value.

A total of 192 KEGG enrichment entries were identified, of which 11 were statistically significant after correction. The most significant pathways included Prostate cancer (q = 0.002422), Proteoglycans in cancer (q = 0.002729), Insulin signaling pathway (q = 0.005716), cAMP signaling pathway (q = 0.007576), Circadian entrainment (q = 0.019175), Insulin secretion, ErbB signaling pathway, Thyroid hormone signaling pathway, ABC transporters, Adrenergic signaling in cardiomyocytes, and Bile secretion. Overall, genes associated with highly differentiated segments are concentrated in networks related to reproductive behavior, endocrine regulation, circadian control, and membrane transport. These cancer-named KEGG entries are interpreted here as shared signaling modules rather than literal disease phenotypes (Table 8 and Table 9, Figure 7).

### 3.9. The Poorly Differentiated Candidate Window Gene Set Provides Only Limited Supporting Evidence

**Figure 8:**
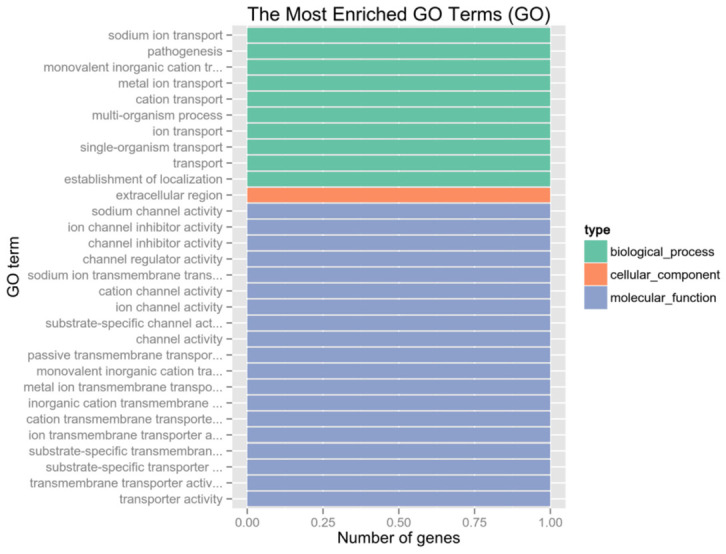
GO enrichment bar chart for the set of candidate genes associated with low differentiation. The enriched items primarily pertain to ion channel activity and transmembrane transport processes.

The poorly differentiated candidate window gene set contains only five windows and three genes; therefore, its enrichment results should be considered only as supplementary observations. In GO, this set primarily involves entries related to ion transport, metal ion transport, cation transport, ion channel activity, and transmembrane transport; in KEGG, only two significant pathways remain: Taste transduction and Inflammatory mediator regulation of TRP channels. Overall, this subset is more oriented toward functions related to membrane receptor sensing and ion channel regulation, but it should not be used to draw independent biological conclusions (Figure 8).

### 3.10. PSMC Reveals That 79 Individuals Share a Similar Deep-Level Demographic History Profile

**Figure 9:**
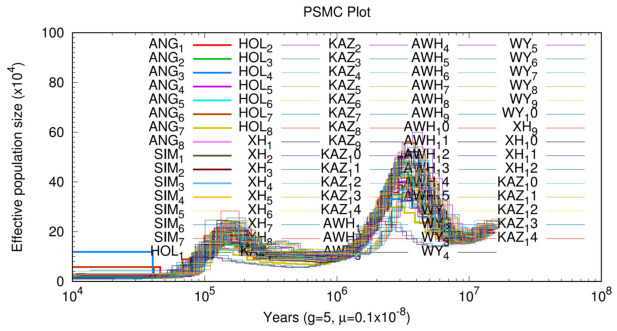
PSMC curves for 79 individuals. The time scale was converted using g = 5 and μ = 0.1 × 10^−8^. These curves are presented only as broad macro-demographic profiles and are not used to infer recent demographic events or subtle between-group differences.

PSMC analysis indicates that 79 individuals share a similar demographic history profile over long time scales and exhibit a common pattern of expansion and decline on a timescale of 10^5^–10^6^ years. Because no bootstrapping was performed and coverage varied among individuals, these curves are retained only as broad macro-demographic profiles and are not used to infer recent demographic change or subtle between-group differences (Table 10, Figure 9).

## 4. Discussion

### 4.1. The Comparative Level of the Study Subjects Dictates That the Main Conclusion Should Be Defined as an “Analysis of Highly Differentiated Segments”

The core comparison in this study is between PTN (69 domestic cattle individuals) and WY (10 yak individuals). In this comparison, the median weighted F_ST reached 0.846. This magnitude primarily indicates deep phylogenetic divergence between domestic cattle and yaks, rather than mild local selection within the same species.

Against this backdrop, candidate high-differentiation windows hold clear research value but are more appropriately interpreted as “locally high-differentiation segments against a background of strong differentiation” rather than being directly equated with recent adaptive sweeps. Relevant methodological studies indicate that F_ST estimates are influenced by the proportion of rare variants and estimation strategies [32], and high F_ST segments may also reflect a decline in local diversity to some extent rather than merely a reduction in historical gene flow [33]; simultaneously, F_ST, π, and Tajima’s D are collectively influenced by population history, population structure, and recombination backgrounds [34,35,36,37,38,39,40,41]. Therefore, the appropriate focus of this study is the analysis of highly divergent segments and candidate functional networks.

### 4.2. Merging the Six Domestic Cattle Populations into PTN Enhanced Statistical Stability but Also Compressed Intra-Breed Variation

Merging ANG, SIM, HOL, XH, KAZ, and AWH into *PTN* substantially increased the domestic cattle sample size and stabilized sliding-window statistics for the primary PTN-WY comparison. This strategy is appropriate for the present biological question because the manuscript is designed to characterize the cross-species background between domestic cattle as a group and yak, not to identify breed-specific selective signals within domestic cattle. Breed-level structure in the same regional system has already been demonstrated by PCA, ADMIXTURE, and neighbor-joining analyses in our recent seven-population study [15], which separated yak, introduced breeds, and local breeds into distinct genetic layers.

However, this merger also compressed internal differences between commercial and local cattle. The Tajima’s D and LD decay patterns of KAZ were markedly different from those of HOL and SIM, indicating that PTN is not a homogeneous background. Candidate highly differentiated segments are therefore interpreted as composite domestic-cattle-versus-yak contrast regions rather than as breed-specific signals. Future work should explicitly compare local cattle versus commercial cattle, local cattle versus yak, and commercial cattle versus yak within the 79-sample framework. In addition, because the sampled ANG, SIM, and HOL animals came from locally maintained commercial herds in Xinjiang rather than from source-country reference panels, the present dataset cannot be used to assert direct genomic equivalence to United States or European populations; any local introgression or adaptation already present in those herds is part of the PTN background.

Furthermore, the act of merging breeds inevitably inflates within-group nucleotide diversity (π_PTN) because it pools the private variations of different breeds. This inflation can affect the π-ratio tails used in our joint scan and may cause weighted F_ST to capture both cross-species divergence and intra-cattle breed structure, rather than purely the former. Downstream, the mean Tajima’s D of PTN is not directly interpretable as a homogeneous population’s frequency spectrum but represents an average across distinct demographic histories (e.g., the strongly negative D in KAZ vs. the positive D in HOL).

### 4.3. The Frequency Spectrum and Linkage Disequilibrium Patterns of Yak Reflect a Long History of Genetic Structuring, Superimposed on Reference Bias

WY exhibited the highest median value and the fewest negative windows for Tajima’s D, and maintained the highest r^2^ for LD decay. This pattern is consistent with a smaller effective population size over the long term, a stronger bottleneck, or a more pronounced history of genetic structuring. The inference that yak have long occupied high-altitude niches is consistent with the overall conclusions of existing yak genome studies [9,10,12,13,14].

It should be noted that all samples were aligned to the Bos taurus reference genome ARS-UCD1.2. This strategy maintains a unified coordinate system but may introduce reference bias in yak through reduced mappability in divergent regions, altered callable-site distributions, locally elevated mismatch rates, downward-biased yak π estimates, inflated F_ST in some windows, and distortion of LD patterns [25]. The consequences of this cross-species mapping bias are systematic and multifold: (i) reduced mappability of yak reads in highly divergent genomic regions leads to systematic missing data; (ii) elevated local mismatch rates can filter out genuine yak variants with lower mapping quality scores; (iii) this results in a downward bias in yak nucleotide diversity (π_WY); (iv) F_ST may be inflated in windows where reference bias is severe, artificially enhancing the apparent divergence; and (v) LD patterns can be distorted due to allelic dropout. Therefore, the extreme statistical characteristics of WY relative to domestic cattle are interpreted as the combined result of true cattle–yak divergence and technical asymmetry intrinsic to cross-species mapping. A yak-specific reference, graph-based reference, or jointly defined callable genome would be valuable in future work.

### 4.4. Functional Enrichment Results Support Differences in Regulatory Networks Rather than Isolated Single-Gene Effects

The GO and KEGG enrichment results for the candidate highly differentiated window sets exhibited distinct thematic clustering. The GO biological process side focused on mating behavior, female receptivity regulation, and reproductive behavior; the KEGG side focused on insulin signaling, cAMP signaling, circadian rhythm, thyroid hormone signaling, and ErbB signaling.

These results suggest that the highly differentiated segments do not focus on isolated structural genes but rather on interconnected regulatory networks. Reproductive behavior, circadian rhythms, energy metabolism, and hormonal regulation are inherently coupled systems. Differences between domestic cattle and yak in life-history rhythms, feeding, and energy allocation are therefore more plausibly discussed at the network level than as the effect of single deterministic loci. This interpretation is consistent with recent systems-oriented views of animal breeding and cattle reproductive genomics [42,43].

Therefore, the functional conclusions of this study should be interpreted at the network level, and individual genes should not be directly defined as determinants of specific traits.

### 4.5. “Cancer Pathways” in KEGG Should Be Interpreted as Shared Signaling Frameworks

The presence of “Prostate cancer” and “Proteoglycans in cancer” among the most significant KEGG entries is not uncommon in population genomics studies of animals. The fundamental reason lies in the fact that KEGG pathways are largely derived from human biomedical pathways, and the core components of these pathways—such as PI3K/AKT, MAPK, RTK, and ErbB—are simultaneously involved in development, metabolism, and cell-to-cell communication.

In this study, such cancer-named pathways were significantly enriched alongside insulin, cAMP, thyroid hormone, and circadian entrainment pathways, indicating that the enriched signal lies in shared regulatory modules rather than disease phenotypes themselves. These entries should therefore be interpreted from the perspective of conserved signaling networks involved in development, endocrine regulation, metabolism, and cell communication [42,43].

### 4.6. Treemix Results Indicate the Presence of Non-Tree-like Relationships

In Treemix models with m = 1–3 migration edges, the residual scale contracted from ±24.1 SE to ±15.7/15.8 SE, indicating that model fit improved when non-tree-like components were allowed. Under the current parameter settings, however, this result is best treated as evidence that covariance among the seven groups cannot be fully explained by a single bifurcating tree, rather than as a quantitative estimate of historical gene flow.

Under the current parameter settings, the primary role of Treemix is to demonstrate that the relationship among Xinjiang domestic cattle and yak cannot be fully explained by a single-branching tree. To evaluate specific admixture scenarios and the direction or magnitude of gene flow, explicit methods such as D-statistics, f4-ratio estimation, or admixture graphs will be required in future work [44].

### 4.7. PSMC Provides a Macro-Level Population History Framework

PSMC curves exhibited similar expansion-recession profiles across all 79 individuals, indicating a shared deep-time demographic background. Because PSMC operates on single diploid genomes, no bootstrapping was performed, sequencing depth varied among samples, and population structure itself can be projected into apparent changes in effective population size [45]; this analysis is retained only as a macro-level historical backdrop. It should not be used to infer recent demographic change or subtle between-group differences.

This treatment aligns the PSMC results with the overall objective of this study, which is to provide broad historical context for the origin of the current highly differentiated pattern rather than to reconstruct recent herd-level demographic events.

### 4.8. Key Conclusions Supported by This Study

This study supports the following conclusions:

(1) There is extremely strong genome-wide divergence between PTN and WY;
(2) Candidate highly differentiated windows form multiple contiguous regions of divergence;
(3) These regions are functionally concentrated in networks related to reproductive behavior, neuroendocrinology, and membrane transport;
(4) Population relationships include non-phylogenetic components and share a deeper demographic history.

## 5. Conclusions

Based on whole-genome resequencing data from 79 individuals, this study characterizes the dominant genomic contrast between a composite domestic cattle population from the Ili River Valley and yak.

The primary signal in the PTN-WY comparison is deep cattle–yak phylogenetic divergence, with multiple locally enhanced highly differentiated segments superimposed on this background. These segments are enriched in interconnected regulatory networks related to reproductive behavior, neuroendocrine regulation, circadian control, and membrane transport, while Treemix and PSMC provide only broad contextual evidence for non-tree-like covariance and shared deep-time demographic profiles.

Overall, the dominant signal confirmed in this study is the deep baseline divergence between cattle and yak. The highly differentiated segments identified here are interpreted as loci of deep species-level divergence rather than recent region-specific adaptive sweeps. Furthermore, given the technical limitations of cross-species mapping bias and the heterogeneity of the composite PTN group, future work employing a yak-specific reference genome and unmerged within-breed comparisons will be necessary to isolate more subtle evolutionary processes.

## Figures and Tables

**Table 1:** Basic Information on Reference Genomes.

Indicators	Value
Seq number	2211
Total length (bp)	2,715,853,792
GC content (%)	41.93
Gap rate (%)	0.00
N50 length (bp)	103,308,737
N90 length (bp)	51,098,607

**Table 2:** Summary of sequencing and alignment statistics for each group.

Group	Sample Size	Raw Data (Gb)	Clean Data (Gb)	Q20 (%)	Q30 (%)	GC (%)	Mapping Rate (%)	Depth (×)	≥1× Coverage (%)	≥4× Coverage (%)
ANG	8	30.88–44.87	30.01–43.88	99.00–99.28	96.15–97.03	44.54–49.40	97.77–99.77	8.23–13.05	82.96–98.27	64.10–91.75
SIM	7	30.38–43.00	29.73–42.41	98.91–99.26	95.71–96.83	43.83–44.77	99.33–99.73	9.59–13.66	98.02–98.68	87.76–97.19
HOL	8	30.15–44.83	29.79–44.26	99.07–99.32	95.82–97.06	43.81–45.27	99.69–99.74	9.34–13.25	98.38–98.56	90.38–96.58
XH	12	20.15–40.08	20.14–38.76	97.54–99.24	93.04–96.93	43.76–44.84	99.07–99.78	5.98–12.30	97.20–98.48	67.42–95.97
KAZ	19	29.53–54.15	27.34–53.73	95.14–99.31	88.93–97.09	43.88–45.42	98.88–99.76	9.47–17.10	97.48–98.64	87.19–97.91
AWH	15	30.14–43.36	29.82–42.83	98.44–99.03	95.22–95.83	44.00–45.05	96.71–99.75	9.81–13.81	98.32–98.63	92.25–97.13
WY	10	35.61–58.13	34.86–57.36	98.92–99.10	96.23–96.66	43.11–44.08	98.86–99.59	10.94–17.26	97.26–97.62	93.43–96.30

**Table 3:** Summary of F_ST sliding-window statistics (MEAN_FST).

Statistic	Value
Total number of windows	132,938
Minimum MEAN_FST	−0.078659
MEAN_FST Q1	0.617864
MEAN_FST	0.705037
MEAN_FST Q3	0.781816
95th percentile of MEAN_FST	0.871117
99th percentile of MEAN_FST	0.945063
Maximum MEAN_FST	1.000000

**Table 4:** Statistics on Candidate Windows+.

Item	Value
Number of candidate highly differentiated windows	832
Number of unique genes in candidate highly differentiated windows	533
Number of low-differentiation candidate windows	5
Number of unique genes in low-differentiation candidate windows	3

**Table 5:** Statistical Summary of the π Sliding Window.

Statistic	Value
Total number of windows	132,332
Minimum PI	0.00000066
PI Q1	0.00022264
Median PI	0.00038477
PI Q3	0.00064950
95th percentile of PI	0.00132583
Maximum PI	0.01111740

**Table 6:** Summary statistics of Tajima’s D across groups.

Group	Number of Windows	Q1	Median	Q3	Proportion of Windows with Negative Values (%)	Proportion of Windows with D > 2 (%)
ANG	66,378	−0.111	0.606	1.266	28.2	6.4
SIM	66,374	−0.289	0.437	1.096	34.0	4.2
HOL	66,272	−0.072	0.695	1.380	27.0	8.8
XH	66,467	−0.363	0.437	1.199	35.7	7.3
KAZ	66,492	−0.429	0.345	1.112	38.4	6.3
AWH	66,508	−0.332	0.443	1.207	35.1	7.6
WY	66,327	0.373	1.173	1.857	17.2	20.3

**Table 7:** Summary of Treemix Model Fits.

Model	Number of Migration Edges	Residual Scale Range
m = 1	1	±24.1 SE
m = 2	2	±15.7 SE
m = 3	3	±15.8 SE

**Table 8:** Top 9 significant GO terms from the candidate set of highly differentiated window genes.

GO ID	Term	Category	Corrected P	Gene Number
GO:0033057	multicellular organismal reproductive behavior	biological_process	2.232 × 10^−6^	9
GO:0044706	multi-multicellular organism process	biological_process	2.232 × 10^−6^	9
GO:0045297	post-mating behavior	biological_process	2.232 × 10^−6^	9
GO:0045924	regulation of female receptivity	biological_process	2.232 × 10^−6^	9
GO:0046008	regulation of female receptivity, post-mating	biological_process	2.232 × 10^−6^	9
GO:0060180	female mating behavior	biological_process	2.232 × 10^−6^	9
GO:0007617	mating behavior	biological_process	2.768 × 10^−6^	9
GO:0007618	mating	biological_process	2.768 × 10^−6^	9
GO:0019098	reproductive behavior	biological_process	2.768 × 10^−6^	9

**Table 9:** Significant KEGG pathways in the candidate set of highly differentiated window genes.

Pathway	Input Number	Background Number	*p*-Value	q-Value
Prostate cancer	16	87	1.295 × 10^−5^	2.422 × 10^−3^
Proteoglycans in cancer	25	201	2.919 × 10^−5^	2.729 × 10^−3^
Insulin signaling pathway	19	140	9.170 × 10^−5^	5.716 × 10^−3^
cAMP signaling pathway	23	199	1.620 × 10^−4^	7.576 × 10^−3^
Circadian entrainment	14	99	5.127 × 10^−4^	1.917 × 10^−2^
Insulin secretion	12	84	1.162 × 10^−3^	3.231 × 10^−2^
ErbB signaling pathway	12	85	1.273 × 10^−3^	3.231 × 10^−2^
Thyroid hormone signaling pathway	14	112	1.511 × 10^−3^	3.231 × 10^−2^
ABC transporters	8	42	1.555 × 10^−3^	3.231 × 10^−2^
Adrenergic signaling in cardiomyocytes	16	146	2.492 × 10^−3^	4.659 × 10^−2^
Bile secretion	10	70	2.914 × 10^−3^	4.953 × 10^−2^

**Table 10:** Parameters and time-scale settings.

Parameter	Setting
PSMC parameters	-N30 -t15 -r5 -p 4 + 25*2 + 4 + 6
Generation time (g)	5
Mutation rate (μ)	0.1 × 10^−8^
Interpretation	Description of broad demographic history

## Data Availability

The raw whole-genome resequencing data analyzed in this study are not publicly available because they are subject to institutional and project-level confidentiality and data-governance restrictions during the current protected period. Supplementary Table S1 provides a reproducibility summary of the preserved workflow parameters and archived output categories that could be directly verified during revision. Processed supporting materials necessary to evaluate the main conclusions are available from the corresponding authors upon reasonable request. Any access to archived raw data is subject to approval by the data-owning institution.

## Supplementary Materials

The following supporting information can be downloaded at: https://www.mdpi.com/article/10.3390/ani16111746/s1, Table S1: Archived Reproducibility Summary for the PTN-WY Revision.

## Abbreviations

ANG	Angus
SIM	Simmental
HOL	Holstein
XH	Xinjiang Brown Cattle
KAZ	Kazakh Cattle
AWH	Altay White-headed Cattle
WY	yak
PTN	composite domestic cat-tle group
LD	linkage disequilibrium
PSMC	Pairwise Sequentially Markovian Coalescent
