# Supplementary material for: Whole-Genome Resequencing Reveals Deep Genomic Differentiation and Highly Differentiated Segments Between a Composite Domestic Cattle Population and Yak from the Ili River Valley and Other Xinjiang Regions
Source: Animals (Basel). 2026 Jun 5;16(11):1746. doi: 10.3390/ani16111746 (PMC13255603; doi:10.3390/ani16111746)
Supplement: Supplementary file 1 [file animals-16-01746-s001.zip › animals-4282205-supplementary.pdf]

Supplementary Table S1. Archived Reproducibility Summary for the PTN-WY Revision

This supplementary file summarizes the workflow parameters and preserved output categories that could be directly verified from the surviving project archive during revision. It also records limitations where original cluster-level logs were no longer recoverable.

Analytical module	Archived, verifiable parameters / inputs	Preserved output status	Notes and limitations
Read quality control	fastp v0.20.0; -g -q 5 -u 50 -n 15 -l 150	Raw/Clean data totals and Q20/Q30 summaries preserved in manuscript Table 2	Per-sample duplicate-rate logs were not recoverable from the surviving archive.
Alignment	BWA-MEM v0.7.17-r1188; mem -t 4 -k 32 -M; SAMtools duplicate removal	Mapping rate, depth, and coverage summaries preserved in manuscript Table 2	Yak was aligned to Bos taurus ARS-UCD1.2 under a unified coordinate system; reference bias may affect cross-species comparisons.
Variant calling / initial screening	bcftools v1.16; mpileup -q 1 -C 50 -a DP,SP,AD -m 2 -F 0.002; loci retained with at least 4 supporting reads and MQ >= 20	Annotated downstream results preserved	The surviving archive does not confirm universal downstream MAF filters, missing-rate filters, biallelic-only pruning, or low-complexity masking across every module.
Sliding-window F_ST and pi scan	Window size 40 kb; step size 20 kb; joint screening based on WEIGHTED_FST and log2(pi_PTN/pi_WY)	Candidate-window counts and summary statistics preserved in the manuscript	Archived screening plot uses WEIGHTED_FST >= 0.93 and extreme pi-ratio tails log2(pi_PTN/pi_WY) <= -1.13 or >= 2.58.
Tajima's D	Window size 40 kb	Group-level summaries preserved in manuscript Table 6	Used descriptively rather than as stand-alone proof of recent selection.
LD decay	PopLDdecay v3.40; -OutStat	LD-decay figure and qualitative ranking preserved	Interpretation remains cautious because LD can also be affected by cross-species reference bias.

Treemix	19,281,978 SNPs; block size = 1000; migration edges m = 1, 2, 3; no outgroup/root specified; no LD pruning	treeout / edges outputs were documented in project delivery records	Used only to show that covariance is not fully explained by a single bifurcating tree; no quantitative admixture claim is made.
PSMC	PSMC v0.6.4-r49; -N30 -t15 -r5 -p 4+25*2+4+6; mpileup -q 1 -C 50; vcf2fq -d 5 -D 200 -Q 20; fq2psmcfa -q10; g = 5; mu = 0.1 x 10^-8; no bootstrap	Original PSMC plot retained and consistent with current manuscript Figure 9	No group-level median curve or bootstrap confidence envelope was preserved in the surviving archive.
Unavailable archived QC metrics	Ti/Tv, pre/post-filter SNP counts for every step, and some cluster-level QC logs	Not recoverable	These values are not reconstructed in the revised manuscript to avoid introducing unsupported claims.
